# Mouse-adapted scrapie strains 139A and ME7 overcome species barrier to induce experimental scrapie in hamsters and changed their pathogenic features

**DOI:** 10.1186/1743-422X-9-63

**Published:** 2012-03-09

**Authors:** Qi Shi, Bao-Yun Zhang, Chen Gao, Jin Zhang, Hui-Ying Jiang, Cao Chen, Jun Han, Xiao-Ping Dong

**Affiliations:** 1 State Key Laboratory for Infectious Disease Prevention and Control, National Institute for Viral Disease Control and Prevention, Chinese Center for Disease Control and Prevention, 155 Changbai Road Changping District, Beijing 102206, People's Republic of China

## Abstract

**Background:**

Transmissible spongiform encephalopathy (TSE) diseases are known to be zoonotic diseases that can infect different kinds of animals. The transmissibility of TSE, like that of other infectious diseases, shows marked species barrier, either being unable to infect heterologous species or difficult to form transmission experimentally. The similarity of the amino acid sequences of PrP among species is believed to be one of the elements in controlling the transmission TSE interspecies. Other factors, such as prion strains and host's microenvironment, may also participate in the process.

**Methods:**

Two mouse-adapted strains 139A and ME7 were cerebrally inoculated to Golden hamsters. Presences of scrapie associate fibril (SAF) and PrP^Sc ^in brains of the infected animals were tested by TEM assays and Western blots dynamically during the incubation periods. The pathogenic features of the novel prions in hamsters, including electrophoretic patterns, glycosylating profiles, immunoreactivities, proteinase K-resistances and conformational stabilities were comparatively evaluated. TSE-related neuropathological changes were assayed by histological examinations.

**Results:**

After long incubation times, mouse-adapted agents 139A and ME7 induced experimental scrapie in hamsters, respectively, showing obvious spongiform degeneration and PrP^Sc ^deposits in brains, especially in cortex regions. SAF and PrP^Sc ^in brains were observed much earlier than the onset of clinical symptoms. The molecular characteristics of the newly-formed PrP^Sc ^in hamsters, 139A-ha and ME7-ha, were obviously distinct from the original mouse agents, however, greatly similar as that of a hamster-adapted scrapie strain 263 K. Although the incubation times and main disease signs of the hamsters of 139A-ha and ME7-ha were different, the pathogenic characteristics and neuropathological changes were highly similar.

**Conclusions:**

This finding concludes that mouse-adapted agents 139A and ME7 change their pathogenic characteristics during the transmission to hamsters. The novel prions in hamsters' brains obtain new molecular properties with hamster-specificity.

## Background

Scrapie, a kind of transmissible neurodegenerative disease in sheep and goats described hundreds years ago, possesses similar neuropathological features and molecular properties as Creutzfeldt-Jakob disease (CJD) and Kuru in human and bovine spongiform encephalopathy (BSE) in cattle. Based on the incubation time, diseases signs, neuropathological characteristics, distribution of PrP^Sc ^in central nerve system, electrophoresis mobility and glycosylation pattern, more than twenty strains of scrapie have been described up to now [1]. Scrapie can transmit horizontally among the sheep and goats, or deliver to the lamb vertically [2]. The infectivity and transmissibility of scrapie, CJD and BSE lead those disorders to being nominated as transmissible spongiform encephalopathies (TSE).

The infectious or pathologic factor of TSE is considered as an abnormal protein without nucleic acid identified so far. The infectious protein, PrP^Sc^, shares the same amino acid sequence as its normal isoform that distributes on the cellular membrane in several kinds cells with GPI-anchor [3]. During the pathogenesis of TSE, PrP^Sc ^may replicate itself, resulting in aggregation to plaque and damage to neuron cells. Probably due to the conformational changes, PrP^Sc ^acquires several particular features once it forms, i.e. insoluble in ordinary degenerates, partially resistant to digestion of protease, resistant to inactivity of UV, radial and commonly used sterilization [4].

TSEs are widely considered as the zoonotic diseases, which can transmit across different species. Historically, the outbreaks of mink spongiform encephalopathy in North America have been believed to be the infection of scrapie from lamb [5]. The most famous example is the outbreaks of BSE in cattle caused by feed of meat-and-bone meal contaminated with scrapie agents, which directly cause emergence of human variant CJD (vCJD) and feline spongiform encephalopathies in cats later [6]. Up to now, more than 220 vCJD cases have been described worldwide, bringing with a great concern in public health. On the other hand, the transmissibility of TSE, like that of other infectious diseases, shows markedly species barrier, either being unable to infect heterologous species, e.g. scrapie of sheep and chronic wasting disease of deer to human, or difficult to form transmission experimentally [7]. It emphasizes again that prion possesses similar general features in transmission across species as other microorganisms, although it has unique biological characteristics.

In the case of housekeeping genes, the genes encoding PrP protein (*PRNP*) are conservative among species during evolution. The mutations within *PRNP *are thought to lead directly to disease without the requirement for an exogenous infectious agent [8]. When an infectious TSE agent transmits to a new host, a specie-barrier has been repeatedly observed, although the mechanism of susceptibility to host has not yet been clearly defined. Polymorphism in PrP from a number of species is thought to play a role in both the TSE host susceptibility and the control of incubation period [9]. The similarities of the amino acid sequences of PrP among species play essential roles in the transmission of prion diseases across animal species. In fact, both naturally-occurred and experimental TSEs show significant tendency to induce the infection more easily onto the species closer to the original one [10].

The emergence of additional novel mammalian prion disease strains has been witnessed since the outbreak of BSE. To get more detailed insight of the alterations of prion disease strains characteristics through interspecies transmission, we inoculated mouse-adapted scrapie strains 139A and ME7 onto Golden hamsters. After different long incubation periods, the experimental TSEs were observed in the inoculated hamsters. We found that the newly-formed strains in hamsters (139A-ha and ME7-ha) obtained new molecular and biochemical features that were similar as that of the hamster-adapted scrapie agent 263 K. Meanwhile, we confirmed again that the appearances of scrapie-associated fibrils (SAF) and proteinase K (PK) resistant PrP in brains infected with agent 139A and ME7 were much earlier than the emergence of clinical manifestations. This finding concludes that mouse-adapted agent 139A and ME7 change their pathogenic characteristics during cross-species transmission in hamsters.

## Results

### Inoculations of mouse-adapted scrapie strains 139A and ME7 into hamsters induced experimental TSE after long incubation

**Figure 1:**
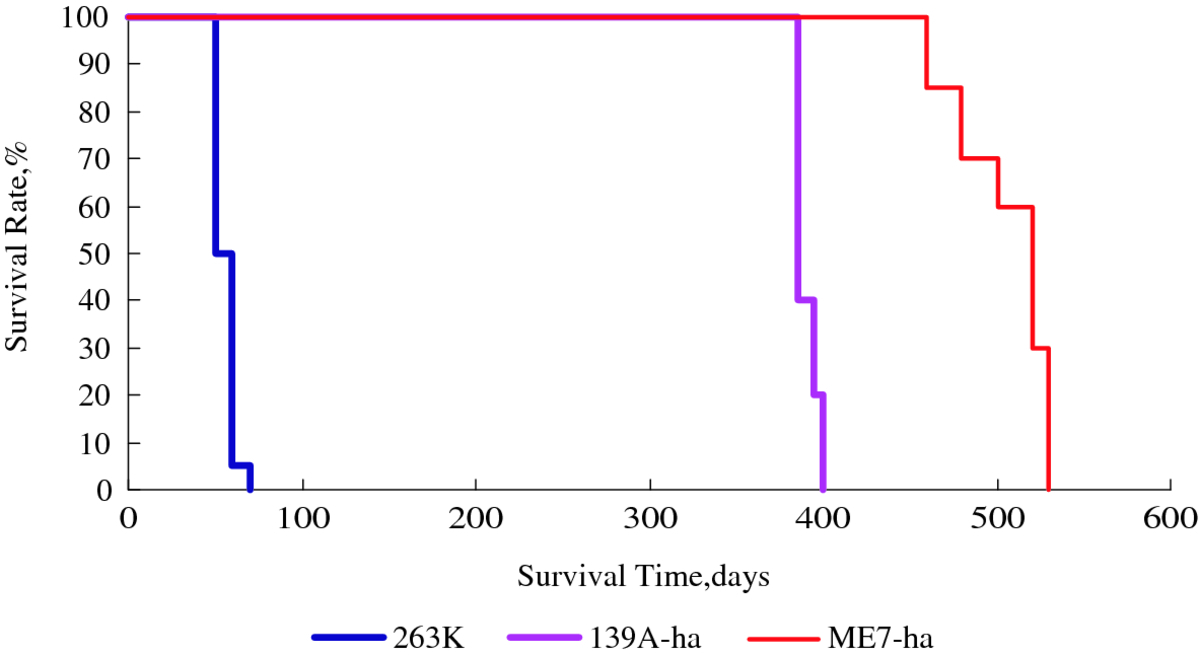
**Comparative incubation periods of the experimental scrapie in hamsters cerebrally infected with the hamster-adapted strain 263 K and mouse-adapted strains 139A and ME7**. The abscissa shows the survival days and the y-axis shows the percentages of the survival.

Brain homogenates of C57 mice infected with mouse-adapted scrapie strains 139A and ME7 were intracerebrally inoculated into hamsters. Except three hamsters in the group of agent 139A died suddenly in the first week after inoculation, all other animals were alive. Animals infected with strain 139A showed the clinical manifestations 385 to 405 days after inoculation, with the average incubation of 395 ± 8.5 days (Figure 1). The most predominant and observable symptoms of the infected hamsters were itchy and scratchy in the later stage, most animals having obvious skin claws. The clinical courses from onset to death persisted from 25 to 30 days (Table 1). Animals infected with strain ME7 became ill 460 to 530 days after inoculation, with the average incubation of 496.25 ± 27.22 days (Figure 1). The clinical manifestations were severe thin and sluggish. The clinical courses of ME7-infected hamsters were 30 to 40 days (Table 1). It confirms again that the mouse-adapted scrapie strains can overcome the species barrier to infected hamsters.

**Table 1:** Comparison of the incubation period, clinical course and main clinical manifestations of the infected hamsters inoculated with three scrapie strains

Strain	Incubation (days)	Clinical course (days)	Clinical manifestation
263 K*	66.7 ± 11 (45-72)	5-7	ataxia

139A	395 ± 8.5 (385-405)	25-30	scratch

ME7	496.25 ± 27.22 (460-530)	30-40	severe thin, sluggish

*referring to [11]

### SAFs and PrP^Sc ^were detected in brain tissues much earlier than the onset of clinical manifestations

Previous study revealed that PrP^Sc ^and SAF appeared almost simultaneously in brains of the hamsters infected with scrapie 263 K earlier than the onset of clinical symptoms [11]. To see the possible appearances in the hamsters' brains infected with scrapie agents 139A and ME7 during incubation period, one animal from each group was randomly selected and the brain was removed surgically 200 days after inoculation, with interval of 20 days till the onset of clinical manifestations. In line with the observation of agent 263 K-infected hamsters, both SAFs and PrP^Sc ^were detected in the brain tissues earlier than the appearance of clinical symptoms. Interestingly, SAFs were observed in brain tissues much earlier than PrP^Sc^, that in the group of agent 139A, SAF was firstly detected in the hamster 224 days, while PrP^Sc ^in the 286 days after inoculation (Table 2), and in the group of agent ME7, SAF was firstly detected in the animal 250 days, while PrP^Sc ^in the 330 days after inoculation (Table 3). Both PrP^Sc ^and SAF were persistently observed in the following tested animals. It indicates that prion-related events, especially SAF, appear much earlier than the onset of clinical manifestations.

**Table 2:** Dynamic analyses of SAF and PrP^Sc ^in the brain tissues of the hamsters infected with scrapie strain 139A

Days after inoculation	200	224	245	264	286	307	328	357	385-405
No. of animals	1	1	1	1	1	1	1	1	5

SAF	-	+	+	+	+	+	+	+	+

PrP^Sc^	-	-	-	-	+	+	+	+	+

Clinical signs	-	-	-	-	-	-	-	-	+

**Table 3:** Dynamic analyses of SAF and PrP^Sc ^in the brain tissues of the hamsters infected with scrapie strain ME7

Days after inoculation	210	230	250	270	290	310	330	350	370	390	460-530
No. of animals	1	1	1	1	1	1	1	1	1	1	6

SAF	-	-	+	+	+	+	+	+	+	+	+

PrP^Sc^	-	-	-	-	-	-	+	+	+	+	+

Clinical signs	-	-	-	-	-	-	-	-	-	-	+

### SAFs of three scrapie strains in hamsters' brains showed the similar morphological structures

**Figure 2:**
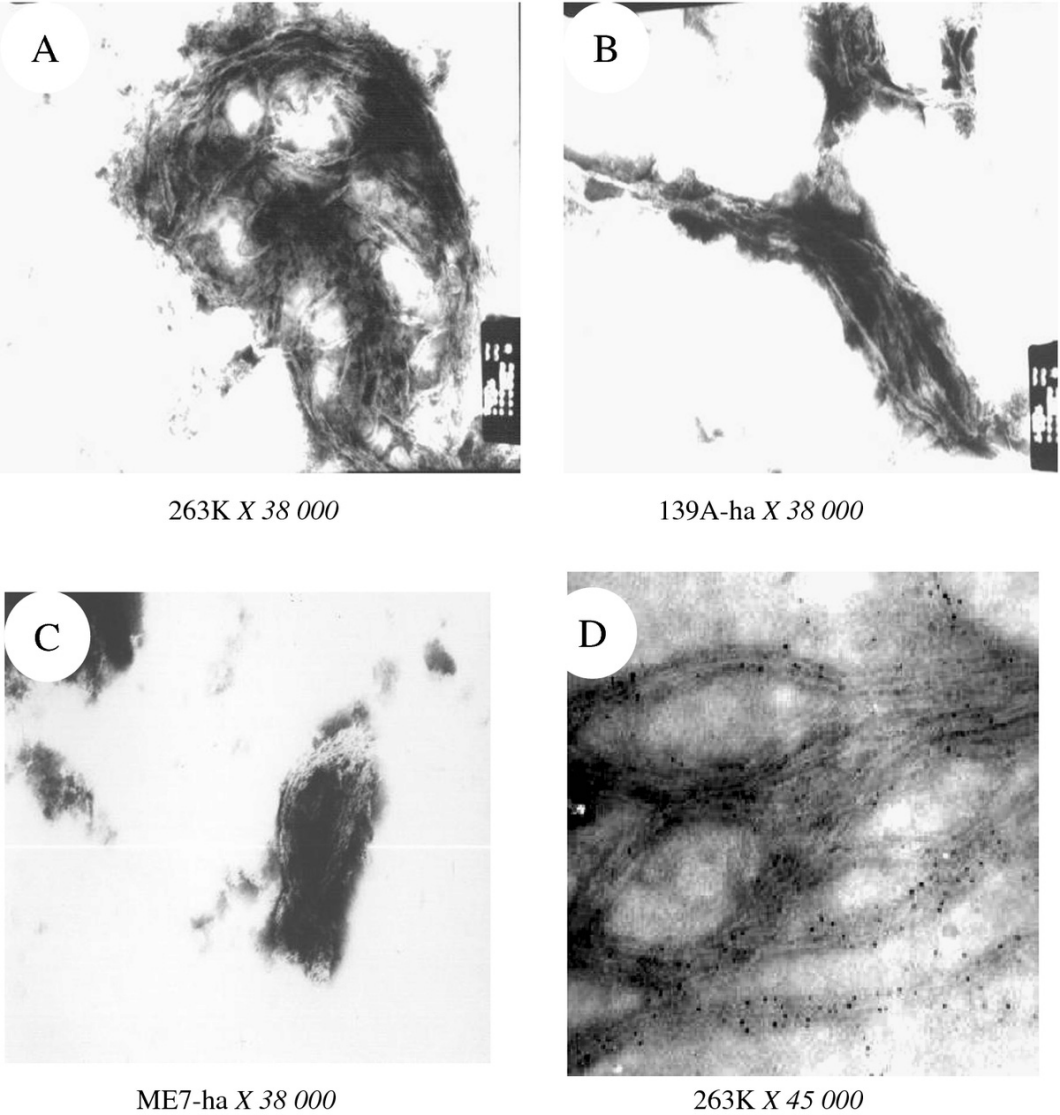
**Morphological assays of SAFs in the hamsters' brains of three different scrapie strains in TEM**. (A) agent 263 K. (B) agent 139A-ha. (C) agent ME7-ha. (D) SAF in the brain homogenates infected with agent 263 K stained by immune colloidal gold technique with PrP specific mAb 3 F4.

Screening more than 5 brain samples of each three strains at the terminal clinical stage identified many long, ramose and roughly 25 nm in diameter fibrils, whose surfaces were shaggy (Figure 2). Compared with the observations of brains infected with agents 263 K (Figure 2A), the amounts of SAFs in 139A-ha (2B) and ME7-ha (2 C) infected hamsters seemed to be smaller, while the SAFs in the ME7-infected brains were relatively shorter. To address the major components of SAFs, brain homogenates were incubated with mAb 3 F4 and subsequently with SPA-coupled gold. EM assays showed large amounts of fibrils covered with black dots (Figure 2D) in the brain homogenates infected with agent 263 K, suggesting that PrP are the main elements of fibrils.

### Similar pathological changes in the brain cortex of the three scrapie strains in hamsters but comparatively severe in the 263 K strain

**Figure 3:**
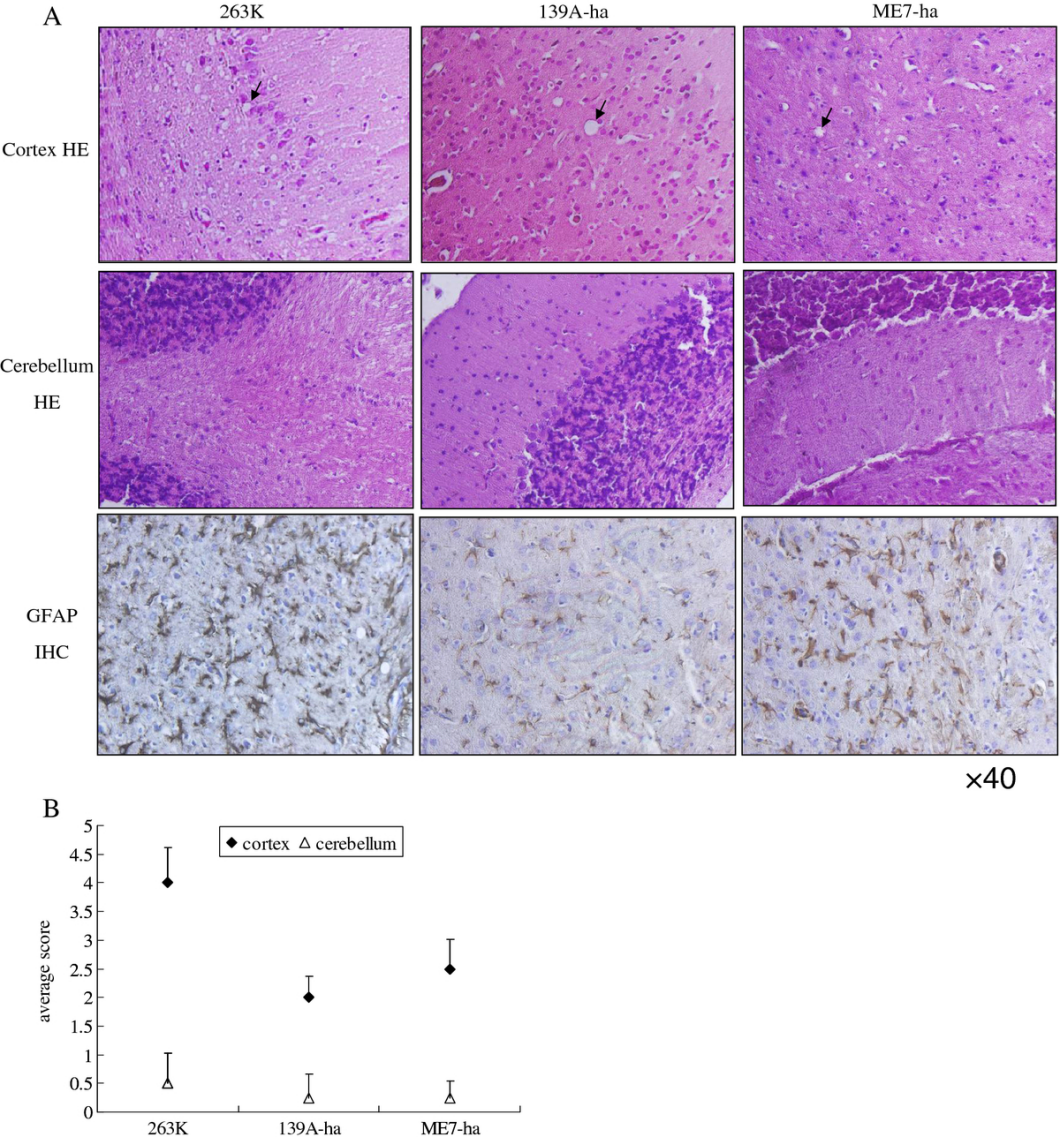
**Neuropathological assays of the regions of cortex and cerebellum from the hamsters infected with agent 263 K, 139A-ha and ME7-ha**. (A) Top and middle panels are HE stains of cortex and cerebellum. Bottom panels are GFAP IHC stains of cortex. The arrows show typical vacuoles. (B) Scores the severity and distribution of vacuolation. The average value for each brain region is calculated from three independent counts and indicated as mean ± SD.

To see the neuropathological characteristics caused by infections with the three scrapie strains in hamsters, histopathological analyses were comparatively performed on the regions of cortex and cerebellum. Spongiform degeneration was more intensive and severe in the cortex infected by strain 263 K, featured with many vacuoles distributed in the field of vision, while moderate spongiform degeneration was observed in the cortex infected by strains 139A and ME7 (Figure 3A, upper panels). The lesion scores of in the cortex regions of strains 263 K, 139A-ha and ME7-ha were evaluated as 4, 2.0 and 2.5, respectively (Figure 3B). Contrast to the changes in the cortex regions, typical spongiform was rarely observed in cerebellum regions of all three strains (Figure 3A, middle panels). Furthermore, gliosis in brain cortex regions were tested by IHC with a commercial mAb against GFAP. Abundant large GFAP-positively stained astrocytes were detected, in which the gliosis in the agent ME7-ha infected brains were comparably severe as that in agent 263 K-infected one, while gliosis in the agent 139A-ha infected brains was significantly mild (Figure 3A, lower panels).

### The PrP^Sc ^molecules of 139A- and ME7-infected hamsters changed their electrophorestic profiles compared with those of their parent mouse-adapted strains

**Figure 4:**
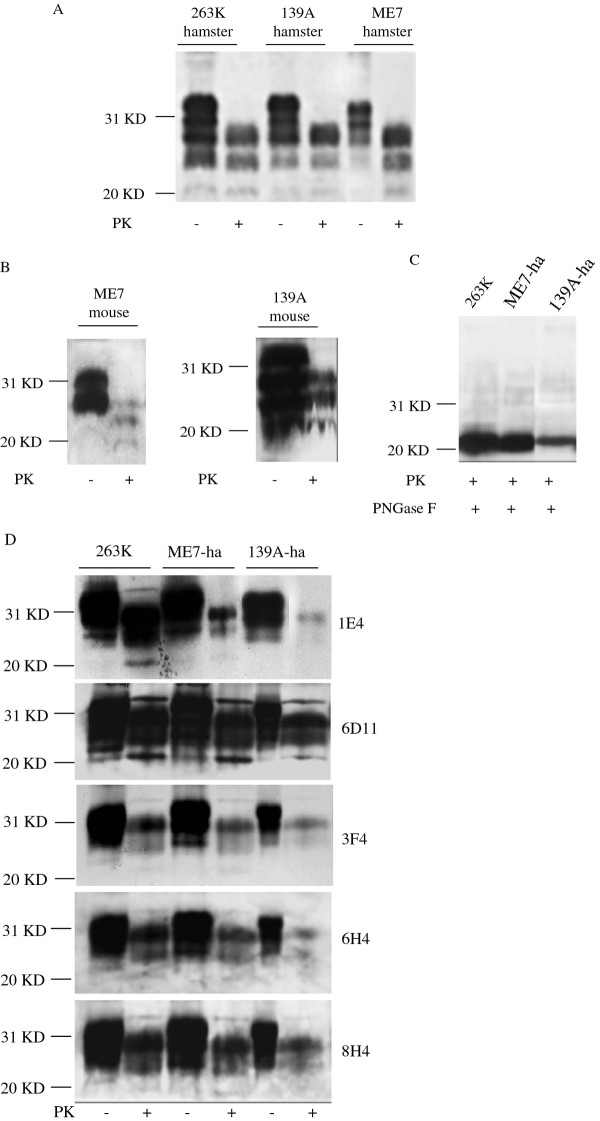
**Analyses for PrP^Sc ^in brain homogenates with Western blots**. (A) PrP^Sc ^in hamsters' brains of agent 263 K, 139A and ME7 detected with mAb 3 F4. (B) PrP^Sc ^in mice brains of agent 139A and ME7 detected with mAb 1E4. (C) Deglycosylated PrP^Sc ^in hamsters' brains of agent 263 K, 139A and ME7 detected with mAb 3 F4. (D) Immunoreactivities of PrP^Sc ^in hamsters' brains of agent 263 K, 139A and ME7 with a panel of PrP specific mAbs, including 1E4, 6D11, 3 F4, 6H4 and 8H4. Molecular weight standards are shown on the left and different mAbs are shown on the right. "+": with PK or PNGase F; "-": without PK The products of PK-digested PrP^Sc ^from the hamsters' brains infected by mouse-adapted agents 139A and ME7 were further exposed to glycosidase. One single PrP-specific bands were observed, which were at the exactly same position in SDS-PAGE as that in the preparation of 263 K PrP^Sc ^(Figure 4C). It implies that the newly-formed PrP^Sc ^in the hamsters' brains by infections of mouse-adapted scrapie agents have the same glycosylated profiles as the hamster-adapted scrapie strain 263 K.

Presences of PrP^Sc ^in brain homogenates of the hamsters infected with strains 139A and ME7 at the terminal stage of diseases were tested by Western blot assays with PrP specific monoclonal antibody 3 F4 after PK treatment. Protease resistant bands (PrP^res^) were seen in all diseased animals, which mobilized roughly at 19-25 kDa. Interestingly, the PrP^res ^from the brains infected with strains 139A and ME7 showed identical electrophoretic and glycosylation profiles, in which the diglycosyl form of PrP^res ^was the most predominant, followed by monoglycosyl and nonglycosyl forms, showing similar profiles as that in the preparation of the hamsters infected with strain 263 K (Figure 4A). Calculating the relative gray values of each glycosyl PrP^Sc ^bands in the reactions with mAb 3 F4 showed that the percentages of diglycosyl, monoglycosyl and nonglycosyl forms in the brains of agent 263 K were 56.8%, 27.9%, 15.3%, the forms of 139A-ha were 52.8%, 32.8%, 14.4% and the forms of ME7-ha were 52.1%, 33.1%, 14.8%. Compared with PrP^res ^in the mice brains infected by the individual parent mouse-adapted strains in Western blot with PrP-specific mAb 1E4, in which the monoglycosyl PrP^res ^were predominant (Figure 4B), the diglycosyl PrP^res ^in hamsters brains of agents 139A and ME7 were predominant (Figure 4A), suggesting that mouse-adapted scrapie strains may change their PrP^Sc ^molecular profiles after replicating in hamsters brains.

To get more information of PrP^res ^profiles, same amounts of hamsters' brain homogenates infected with three different scrapie agents at the terminal stage of diseases were comparatively assayed by Western blots with other commercial PrP-specific mAbs, including 1E4, 6D11, 6H4 and 8H4. Like that in the reactions with mAb 3 F4, similar electrophoretic and glycosylated PrP^res ^patterns were observed in the preparations reacted with the tested antibodies, all showing predominately diglycosal PrP^res ^(Figure 4D). In line with the observations in Figure 4A reacted with mAb 3 F4, clear small molecule-weight bands at the position of *Mr *21 were detected in all three homogenates in the reaction with mAb 6D11, indicating presences of the truncated C2 fragments (Figure 4D). Additionally, at our experimental condition, relatively weaker signals of PrP^res ^were observed in 139A infected hamsters' brains in the Western blots with various PrP mAbs.

### The PrP^Sc ^of the three scrapie strains in hamsters had the similar PK-resistance

**Figure 5:**
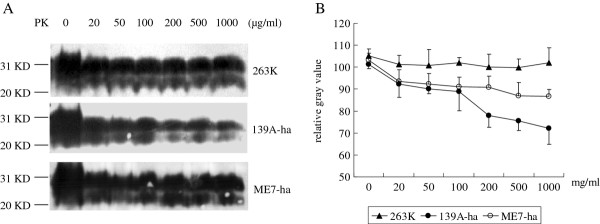
**PK resistance of PrP^Sc ^in hamsters' brains of agent 263 K, 139A-ha and ME7-ha**. (A) Western blots with mAb 3 F4. The concentrations of PK in individual preparation are indicated on the top. Molecular weight standards are shown on the left. (B) Quantitative analyses of each gray numerical value of PrP blots. Relative gray values of the PrP signals in each experiment are normalized by division with that of the respective reaction without PK. The average values are calculated from three independent blots and presented with as mean ± SD.

To test the PK-resistances of PrP^Sc ^from the three scrapie strains, brain homogenates from 3 infected animals at the terminal stage of diseases were pooled as the representative samples and exposed to the digestion with different amounts of PK from 20 to 1000 μg/ml. The input PrP amounts in various homogenates were equilibrated by Western blots prior to PK-treatment. Figure 5A revealed PrP^res ^signals in all preparations at the experimental condition. Compared with the input PrP, the intensities of PrP^res ^signals of the three scrapie strains in the preparations of 20 μg/ml PK decreased slightly. The PrP^res ^signals of scrapie agents 263 K and ME7 remained fairly stable in the reactions containing 20 to 1000 μg/ml PK, but that of agent 139A started to drop down from the preparation of 200 μg/ml PK (Figure 5B). These results indicate that the PrP^Sc ^in hamsters' brains by infections of hamster-adapted agent 263 K or mouse-adapted agents 139A and ME7 have similar PK-resistant features.

### The PrP^Sc ^of the three scrapie strains in hamsters had similar conformational stability

**Figure 6:**
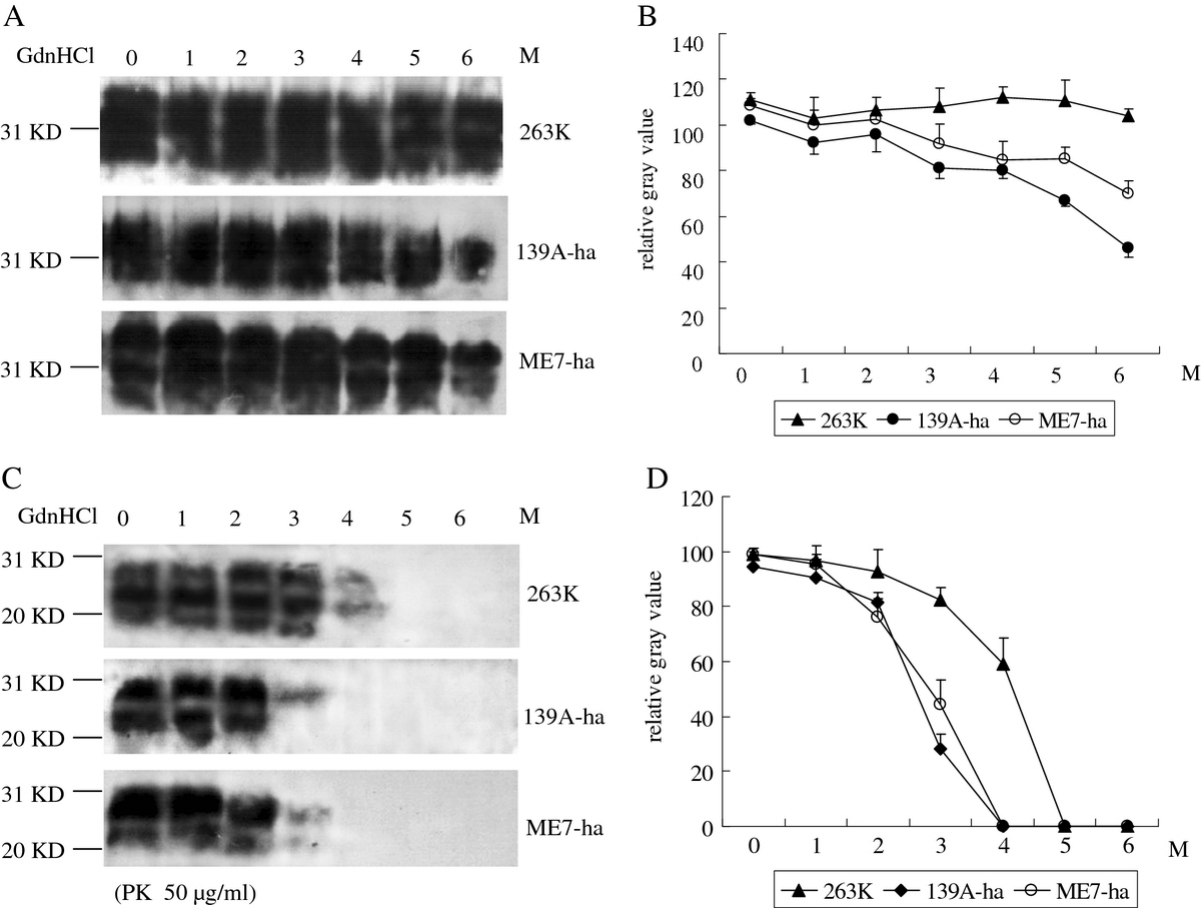
**Conformational stability of PrP^Sc ^in hamsters' brains of agent 263 K, 139A-ha and ME7-ha**. (A) Western blots of total PrP with mAb 3 F4. Hamsters' brain homogenates are incubated with 1 to 6 M GdnHCl. The concentrations of GdnHCl in individual preparation are indicated on the top. Molecular weight standards are shown on the left. (B) Quantitative analyses of each gray numerical value of total PrP blots. The average values are calculated from three independent blots and presented with as mean ± SD. (C) Western blots of PrP^Sc ^with mAb 3F4. Hamsters' brain homogenates are incubated with 1 to 6 M GdnHCl and subsequently exposed to 50 μg/ml PK. The concentrations of GdnHCl in individual preparation are indicated on the top. Molecular weight standards are shown on the left. (D) Quantitative analyses of each gray numerical value of PrP^Sc ^blots. The average values are calculated from three independent blots and presented with as mean ± SD.

Exposure of PrP^Sc ^to increasing concentrations of GdnHCl leads to a transition from the native to denatured state, measured as loss of resistance to protease digestion. To test the consistency of three kinds of PrP^Sc ^in the PK-resistance after exposing to GdnHCl, the representative samples of these three infected hamsters were incubated with a series of amounts of GdnHCl from 1 to 6 M separately. The input PrP amounts in various homogenates were equilibrated by Western blots prior to GdnHCl- treatment. Western blots with mAb 3 F4 revealed that the total PrP signals from preparations of the agent 263 K remained comparatively constant among the reactions with different concentrations of GdnHCl while PrP signals of the agents 139A-ha and ME7-ha weakened clearly only in the reactions of 6 M GdnHCl (Figure 6A and 6B). The GdnHCl-treated products were subsequently exposed to the proteolysis with 50 μg/ml PK. PrP^res ^signals were detectable in preparations of the agents 139-ha and ME7-ha incubated with GdnHCl below 4 M and in that of the agent 263 K incubated with GdnHCl below 5 M (Figure 6C). Calculations of the relative gray values of PrP^res ^signals showed that PrP^Sc ^in the preparations of agents 139A-ha and ME7-ha started to drop down remarkably after exposed with 3 M GdnHCl and disappeared since 4 M GdnHCl, whereas PrP^Sc ^of agent 263 K became notable weak after exposed with 4 M GdnHCl and undetectable since 5 M GdnHCl (Figure 6D). These data indicate that the mouse-adapted scrapie strains 139A- and ME7-induced PrP^Sc ^in the brains of hamsters have the similar conformational stabilities as that of the agent 263 K-induced PrP^Sc^.

**Figure 7:**
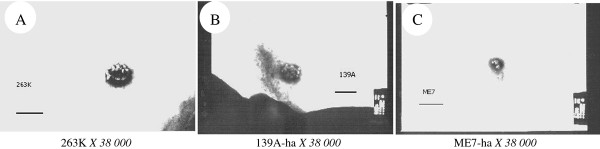
**Morphological assays of SAFs in the hamsters' brains of three different scrapie strains after incubated with 3 M GdnHCl**. (A) agent 263 K; (B) agent 139A-ha; (C) agent ME7-ha.

To see the potential influences of GdnHCl on the morphology of SAFs, brain homogenates of 263 K, 139A-ha and ME7-ha-infected hamsters were individually incubated with 3 M GdnHCl. EM assays identified the clusters of small round particles and short clubs stained by phosphomolybdic acid in the three scrapie strains (Figure 7). No SAF-like structures with long, ramose or straight fibrils were observed after exposing to GdnHCl.

## Discussion

In this study, we have set up two experimental scrapie infections on hamsters by cerebral inoculations of mouse-adapted scrapie agent 139A and ME7. Typical neuropathological abnormalities of TSE and deposits of PrP^Sc ^have been observed in the brains of the infected hamsters, confirming again that mouse-adapted scrapie agent can overcome species barrier to infect hamsters. The PrP proteins of mouse and hamster share a great deal of homology in amino acids sequences and in tertiary structures, with only seven amino acids differences. Our data here provide the evidences that hamster's PrP^C ^supplies as the substrate for replication of mouse-derived PrP^Sc^. A number of sCJD strains can transmit more efficiently to the human PrP transgenic mouse lines than to wild type mice, which shows shorter incubation times and higher susceptibility [12]. However, it makes an exception that replacement of the murine PrP gene with bovine PrP gene led surprisingly to longer incubation period for BSE in the transgenic mice than in the wild type mice despite the increase in identity between the host and donor PrP [13]. It seems that except for the consistence of *PRNP *sequences, other unknown factors will affect on the host susceptibility. Like other TSE transmissions among different species [14], the clinical manifestations in the infected hamsters emerge extremely late after long incubation times. It reflects an inefficient conversion from hamster's PrP^C ^to PrP^Sc ^by exotic mouse prion protein during the first passage. Successive passages of the new strains in hamsters in future may decrease and fix the respective incubation periods.

Different incubation times and clinical courses in hamsters by infection of agents 139A and ME7 imply that besides the amino acid homology between mouse and hamster, scrapie strain is another element for the transmission across species. Such phenomena have been described elsewhere [15]. The amounts of the infectious agents in this study seem not to be the essential reason, since the levels of PrP^Sc ^of two strains, regardless in mice brains as the inoculum or in hamster brains as the product, are quite comparable. One speculation may lie on the differences in their unknown tertiary structures of those two prion strains, leading to the differences possibly in molecular level during conversion from PrP^C ^to PrP^Sc^.

The hamsters infected with mouse-scrapie agent 139A and ME7 possess similar pathogenic and pathological changes. Instead of the predominantly monoglycosyl PrP^res ^in the original mouse-adapted strains, the PrP^res ^formed in the hamster brains infected with agents 139A and ME7 are predominant diglycosylated, which show the same glycosylation patterns as that of a hamster-adapted scrapie strain 263 K. Apart from the glycosylating profiles of PrP^Sc^, other main biochemical features of the two newly-formed PrP^Sc ^in hamsters, i.e. immunoreactivity, PK-resistance, solubility and stability in GdnHCl, are highly comparable with that of agent 263 K. Those data indicate that two kinds of the newly-formed PrP^Sc ^in hamster brains lose their original molecular characteristics in mouse brains, while obtain new properties that show markedly hamster-specific.

Our data emphasize the host microenvironment affects obviously the molecular features of the new PrP^Sc ^generated during transmission across species. Other previous studies have shown that TSE strains alter their characteristics during the passage in a foreign species and the changed features maintained stably with the serial passage, besides the latent period became shorter than the first passage [16,17]. It is belived that inter-species prion disease transmission is frequently the acquisition of new strain properties, in which transgenic mouse and protein misfolding cyclic amplification (PMCA) approaches provide a facile means of generating and characterizing novel prion strains [18]. However, some kinds of prion strains never sacrifice the original molecular properties when infecting onto other species. The famous example is BSE agent, which keeps its main biochemical and molecular characteristics after causing infection on human (vCJD), cats (FSE) and other ungulates (exotic ungulate encephalopathy) [19]. The exact reason for such difference among prion strains remains unclear. It is speculated that interactions with chaperones or other cellular factors, depending on prion variant, will be at least part of some species barriers [20]. Nevertheless, this mysterious phenomenon lies at least on prion strain, host PrP^C ^and host microenvironment.

During the course of TSE, a kind of protein fibril, referred as SAF, is usually observed in brain tissues [21]. SAFs are abnormal structures uniquely associated with prion diseases of many species. The major, even exclusive component for SAF is PrP^Sc ^[22]. The structure of SAF *in vitro *can be destroyed easily by many physical and chemical agents, including GdnHCl in this study. Although it is still not settled whether the infection of prion needs a fixed morphological structure like virus, it is certain that maintenance of SAF in inoculum is not indispensable. Our previous study [23] and others [24] have repeatedly addressed GdnHCl-treated brain extracts from scrapie infected animals, in which the fibriform structures of SAFs are undetectable, still maintain its infectivity. Unfortunately, the SAF structures of the original mouse-adapted strains 139A and ME7 are hard to be observed, possibly because of long-term stored specimen. Comparison of the SAF structures of the one TSE agent from different infected species will help to understand the potential ultra-structural changes in during interspecies transmission.

One of the features of prion diseases is that they usually have extremely long incubation. In line with the results of our previous study of the experimental bioassays on hamsters with scrapie agent 263 K [11], SAF and PrP^Sc ^have been observed in brains infected with agent 139A and ME7 during their incubation periods, which are much earlier than the appearance of clinical symptoms. Those accord well with the natural phenomenon of almost all infectious diseases that the pathogens are usually detectable earlier than the appearance of clinical symptom. Interestingly, SAFs are observed much earlier than the PrP^Sc ^in the brain tissues from the two infections in our experimental condition. Whether it is a general feature for TSEs is not known. If it were, it would highlight that the nascent constructs of PrP^Sc ^in the early stage of disease may not be stable enough for resisting the routinely concentrated PK digestion. Nevertheless, our data stress again that assays for SAF and PrP^Sc ^in brains are useful biomarkers for screening TSE before onset of symptoms.

Conformational stability of PrP^res ^has also been used to differentiate TSE strains. When PrP^C ^converts to PrP^Sc^, the increased component of β-sheet structure makes the prion protein more stable to resist the effectiveness of GdnHCl and PK [25]. According to our data, the resistibility of the three hamster-adapted scrapie strains to GdnHCl and PK is almost similar, though strain 139A-ha is slightly weak. This similarity elucidates that a consistency of PrP^res ^form in hamsters in the molecular level.

## Methods

### Animal bioassay

Brain materials from hamster scrapie strain 263 K, mouse scrapie strains 139A and ME7 were homogenized (1:10) prior to challenging as inoculum. 1 μl of individual brain homogenate was intracerebrally injected into 15-day old Golden hamsters under halothane anaesthesia respectively. Each group consisted of 16 hamsters. For equilibrating the injected amounts of the different strains, we analyzed the absolute PrP^Sc ^grey value of different strains by Western blot method with the same loading volume after totally PK digestion. The clinical symptoms and signs were scored as described previously [11] and the incubation was calculated from the inoculation to the terminal stage of the disease individually. Then the animals were euthanized at the end of clinical phase and brains were taken surgically for further studies during the incubation period and after the onset of illness.

### Transmission electron microscopy (TEM) assays

The individual brain homogenate of hamster scrapie strain 263 K, hamster scrapie strains 139A and ME7 were absorbed onto copper nets covered with carbon membrane and stained with 2% phosphomolybdic acid for 2 min at room temperature. Scrapie-associated fibril (SAF) was observed with transmitted electron microscopy at the condition of 80 KV (Philips, JEOL1200EX). For colloidal gold immunoelectron microscopy assays, samples were absorbed onto copper nets and incubated with 1:100 diluted mAb 3 F4 for 8 hr. After washed with PBS for three times, the copper nets were exposed to 1:50 diluted 5 nm SPA-immunogold for 1 hr and stained with 2% phosphomolybdic acid as described above.

### Pathological assays

Brain tissues of different hamster-adapted strains were fixed in 10% buffered formalin solution. Before histological processes, all the fixed tissues were immersed in 98% formic acid for at least 1 h for inactivation. Paraffin sections (5 μm in thickness) were subjected to conventional staining with hematoxylin and eosin (HE). The spongiform degeneration for the three strains was monitored by light-microscopy and the severity and distribution of vacuolation were measured according to the protocol described elsewhere [26], briefly, 0, no lesions; 0.5, minimum vacuolation (2-3 vacuoles in half a × 40 objective field); 1.0, little vacuolation (3-5 vacuoles in half a field); 2.0, moderate vacuolation (several vacuoles evenly scattered); 3.0, extensive vacuolation (many vacuoles distributed in half a field); 4.0, severe vacuolation (numerous vacuoles often coalescing). For glial fibrillary acidic protein (GFAP), the sections were incubated with 1:500 diluted anti-GFAP mAb at 4°C overnight. Subsequently, the goat anti-mouse IgG biotinylated antibody diluted 1/200 in 10% normal goat serum was incubated for 30 min at room temperature, and an avidin-biotin-peroxidase complex was applied using diaminobenzidine (DAB) as a substrate. Finally, sections were counterstained with hematoxylin for 1 min, dehy-drated, and routinely mounted [11].

### Purification of PrP^Sc ^and proteinase K (PK) digestion

The brain samples of the infected hamsters and mice were homogenized in 10% lysis buffer (100 mM NaCl, 10 mM EDTA, 0.5% Nonidet P-40, 0.5% sodium deoxycholate, 10 mM Tris, pH 7.5) according to the protocol described elsewhere [27]. PrP^Sc^-enriched fractions of brain homogenate prepared from the different strains were centrifuged at 1000 rpm for 10 min and the cell debris was removed. The supernatants were collected and centrifuged at 20 000 g for 90 min, and the pellets were treated again as above. Then the pellets were mixed and stored at -80°C as the purified insoluble PrP^Sc^.

The brain homogenates from three biological samples of each strain collected at the terminal stage were pooled as the representative samples of individual strains. For detection of PK-resistant PrP (PrP^res^) in brain tissues, the samples were incubated with 50 μg/ml of PK (Merck) at 37°C for 60 min. For evaluation of PK-resistances of PrP^Sc ^from various hamster-adapted strains, the brain specimens were treated with different amounts of PK at the final concentrations of 20, 50, 100, 200, 500 and 1000 μg/ml at 37°C for 60 min. The digestions were stopped with 3 mM PMSF (Sigma) for Western blot. Different strain samples were undergone with 3 separate PK treatments.

### Western blots

Samples were separated in 12% SDS-PAGE and electronically transferred to a nitrocellulose membrane according to the protocol described elsewhere [28]. For the mouse-derived specimen, PrP-specific monoclonal antibody (mAb) 1E4 (dilution 1:1000) was used as the primary antibody. For the hamster-derived samples, PrP-specific mAb 1E4, 6D11, 3 F4, 6H4 and 8H4 (dilution 1:1000) were used respectively. The reactions were conducted in TBS-T (10 mM Tris-HCl, pH 7.8, 100 mM NaCl, 0.05% Tween 20) containing 5% (wt/vol) nonfat milk at 4°C overnight and subsequently washed three times with TBS-T. Then the membranes were incubated with horseradish peroxidase-conjugated (HRP)-conjugated goat anti-mouse immunoglobulin G (Santa Cruz) at 37°C for 1 h and PrP-specific signals was detected with an ECL detection kit (Amersham-Pharmacia Biotech).

### Deglycosylation assay

After mixed with equal volume of glycoprotein denaturing buffer (New England Biolabs), various PrP^Sc ^preparations were heated at 100°C for 10 min. Subsequently, 50 mM sodium phosphate, pH 7.5, containing 1% NP-40 and 2 μl of N-glycosidase F (1,800,000 U/mg, New England Biolabs) were added into the samples and the mixtures were incubated at 37°C for 2 h. PrP signals in each preparation were detected by Western blot as described above.

### Conformational stability assay

100 μl of the representative sample each strain mentioned above were mixed with five volumes of cold methanol at -20°C for 2 h. After centrifuged at 20 000 g for 30 min, the pellets were resuspended with 100 μl of different concentration of GdnHCl, including 1, 2, 3, 4, 5 and 6 M and incubated at 37°C for 12 h as described elsewhere [29]. Subsequently, five volumes of cold methanol was added into each preparation and maintained at -20°C for 2 h. After centrifuged at 20 000 g for 30 min, the pellet was resuspended with 100 μl TN buffer (10 mM Tris, 130 mM NaCl, pH 7.0). Half part was employed directly into SDS-PAGE and the rest was subjected into PK-digestion (50 μg/ml) at 37°C for 1 h Different strain samples were undergone with 3 separate GdnHCl and PK treatments.

### Quantitative and statistical analysis

Quantitative analysis of immunoblot images was carried out using computer-assisted software Image Total Tech (Pharmacia). Briefly, the image of immunoblot was scanned with Typhoon (Pharmacia) and digitalized, saved as TIF format. The values of each target blot were evaluated. All data are presented as the mean ± SD.

## Conclusions

The paper concludes that mouse-adapted agent 139A and ME7 change their pathogenic characteristics during interspecies transmission on hamsters. The novel prion strains formed in hamsters' brains obtain new molecular properties with hamster-specificity.

## Ethical approval

This study was approved by the Ethical Committee of National Institute for Viral Disease Prevention and Control, China CDC.

## Competing interests

The authors declare that they have no competing interests.

## Authors' contributions

QS carried out all experiments in this study, collated the information, performed the literature search and drafted the manuscript. BYZ and CG assisted to perform the neuropathological assays and animal tests. JZ performed the animal experiment. HYJ, CC and JH assisted to finish the Western blots. XPD, the corresponding author, designed the research project, performed the literature search and prepared the manuscript. All authors read and approved the final manuscript.
